# Metal-Functionalized Nanozymes in Antibacterial Wound Management: Recent Advances and Future Perspectives

**DOI:** 10.3390/ph19020333

**Published:** 2026-02-19

**Authors:** Selvam Sathiyavimal, Devaraj Bharathi, Ezhaveni Sathiyamoorthi

**Affiliations:** 1 Oral and Maxillofacial Surgery and Digital Implant Surgery Research Unit, Chulalongkorn University, Bangkok 10330, Thailand; sathiyavimal.s@chula.ac.th; 2 School of Chemical Engineering, Yeungnam University, 280 Daehak-Ro, Gyeongsan 38541, Republic of Korea; devarajbharathi@yu.ac.kr

**Keywords:** nanozymes, metal functionalization, antibacterial mechanisms, wound-healing applications

## Abstract

Chronic and infected wounds continue to pose significant clinical challenges due to microbial infections, biofilm development, inflammation, and poor tissue regeneration. Traditional antibiotics medications often show low efficacy and lack stability. The demand for new therapeutic approaches is increasing due to bacterial resistance. Metal-based nanozymes have intrinsic enzyme-like catalytic activity and emerged as a promising class of antibacterial agents for wound-healing applications. The functionalization with metals such as silver (Ag), copper (Cu), iron (Fe), manganese (Mn), cerium (Ce), platinum (Pt) and gold (Au) enhances peroxidase (POD)-, oxidase (OXD)-, and catalase (CAT)-like biomimetic activities. This improvement enables efficient reactive oxygen species (ROS) production, biofilm inhibition, and microenvironment-responsive antibacterial activity. These metal-nanozymes also alter the immune response, increase angiogenesis, and promote extracellular matrix remodeling when combined with metals and also polysaccharides. This review summarizes recent advances in metal-incorporated antibacterial nanozymes including their design, catalytic mechanisms, structure–activity relationships, and integration into hydrogels, films, and fibers for wound healing. Key challenges such as biosafety, metal ion release, the inflammatory balance, and clinical translation are critically discussed. Emerging directions such as single-atom nanozymes, cascade enzyme systems, and stimuli-responsive platforms are highlighted as promising routes for next-generation wound therapeutics. Overall, this review underscores the clinical potential of metal-functionalized nanozymes for infected wound management; however, concerns regarding ion leakage and long-term safety persist emphasizing the need for controlled designs and biocompatible systems to enable safe translation.

## 1. Introduction

Acute and chronic wounds affect millions globally, leading to increased morbidity, mortality, and healthcare costs [1,2]. Impaired wound healing is prevalent in diabetic patients and is associated with prolonged inflammation, insufficient oxygenation, and delayed tissue regeneration [3,4]. One of the main factors preventing wound regeneration is the increased risk of infection, especially by multi-drug resistant (MDR) pathogens that readily colonize open wounds and form strong biofilms [5,6]. Bacteria can become up to several times more resistant to drugs as a result of these biofilms acting as barriers [7]. In addition, they impede the natural healing cascade by causing inadequate angiogenesis, elevated oxidative stress, and persistent inflammation.

Significant drawbacks of conventional antimicrobial drugs include weakening efficacy, poor stability in biological microenvironments, and a concerning rise in antibiotic resistance [8]. These issues underscore the urgent need for new therapeutic approaches that can provide multimodal antibacterial, antioxidant, and regenerative advantages. In this regard, nanozymes that naturally mimic enzymes have become a potential next-generation solution [9,10]. Nanomaterials with enzyme-like activity are commonly referred to as nanozymes and were originally developed as synthetic catalysts. Nanozymes have quickly evolved into powerful biomedical tools due to their notable stability, mixed catalytic capabilities, and ability to operate in physiological conditions, where natural enzymes typically lose activity [11,12].

**Figure 1:**
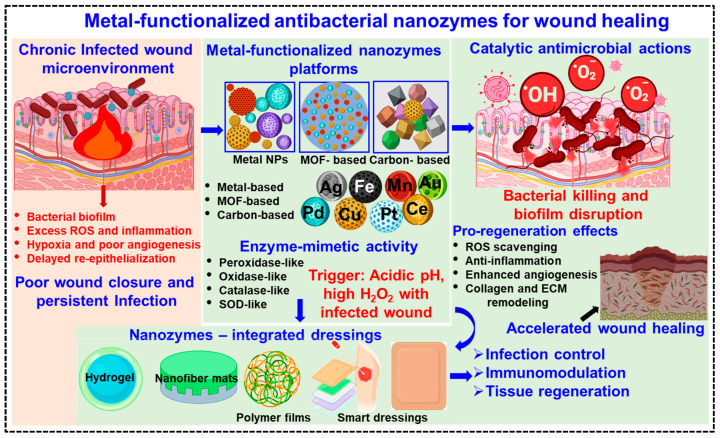
Schematic diagram representing the antibacterial wound-healing activity of nanozymes. Metal-functionalized nanozymes exhibit enzyme-mimetic catalytic activity in infected wound environments, leading to bacterial killing, biofilm disruption, ROS regulation, immunomodulation, enhanced angiogenesis, extracellular matrix (ECM) remodeling, and accelerated wound healing.

Among the various types of nanozymes for applications in wound healing, metal-functionalized antibacterial nanozymes have attracted much attention. The incorporation of metals such as silver (Ag), copper (Cu), iron (Fe), manganese (Mn), cerium (Ce), platinum (Pt), palladium (Pd) and gold (Au) plays a central role in defining the catalytic and antibacterial performance of nanozymes. These metals provide accessible redox states that enable peroxidase (POD)-, oxidase (OXD)-, and catalase (CAT)-like activities, leading to efficient ROS generation, biofilm disruption, and broad-spectrum antibacterial action [13,14]. In addition to catalytic effects, the controlled release of metal ions (e.g., Ag^+^, Cu^2+^, Fe^2+^/Fe^3+^) further enhances antibacterial efficacy by interfering with bacterial metabolic pathways and membrane integrity. Owing to their high physicochemical stability, tunable redox behavior, and adaptability to the wound microenvironment, metal-based nanozymes exhibit greater robustness and sustained activity compared with natural enzymes, making them highly suitable for long-term wound-healing applications [15]. An overview of metal-functionalized antibacterial nanozyme mediated wound healing is shown in Figure 1.

Based on recent growing interest in catalytic materials for wound care, this study addresses the lack of an integrated, mechanism-oriented understanding of how metal-functionalized nanozymes simultaneously achieve antibacterial efficacy and support wound healing. Unlike existing reviews [16,17,18], this review provides a unified and up-to-date (2023–2025) overview of metal-functionalized antibacterial nanozymes, integrating classification, catalytic mechanisms, structure–activity relationships, and therapeutic applications, while emphasizing recent advances such as stimuli-responsive wound dressings, cascade catalytic systems, and single-atom nanozymes (SAzymes), along with key biosafety and translational considerations.

## 2. Fundamentals of Nanozymes

Nanozymes are substances that have the ability to mimic the catalytic functions of natural enzymes [19]. They exhibit high stability, tunable activity, low production costs, and functionality under physiologically demanding conditions. Consequently, they have garnered significant attention in the field of biomedicine since their discovery. Nanozymes are ideal for treating chronic or infected wounds because they give a clear advantage in wound healing through catalytic antibacterial activity, the control of ROS, and their ability to break up biofilms [20]. Their size, surface properties, composition, and structure all influence their enzyme-mimetic behavior, which in turn influences their catalytic activity [21]. These characteristics enable the broad categorization of nanozymes into several important classes. The following subsections provide detailed categories of nanozymes.

### 2.1. Types of Nanozymes

#### 2.1.1. Metal Nanozymes

Metal nanozymes are among the most researched classes due to their high catalytic efficiency and strong redox enzyme mimetic activity. Compared with metal-free nanozymes, such as carbon- or polymer-based systems, metal nanozymes offer superior redox flexibility and higher catalytic efficiency owing to their multiple accessible oxidation states. Ag [22], Au [23], Pt [24], Cu [25], Fe [26], Mn [27], and other metals have intrinsic enzyme-mimetic activity. Their catalytic capabilities, which stem from electron-transfer activities on the metal surface, enable rapid ROS production for antibacterial activity. Furthermore, the higher degree of tunability of metal nanozymes allows for surface functionalization, alloying, and size modification to improve catalytic efficiency and biocompatibility [28,29]. For example, Wang et al. [30] developed Cu single-atom-doped porous carbon nanozymes with uniformly dispersed Cu sites. These nanozymes exhibited enhanced POD-like activity, glutathione depletion, and photothermal effects, leading to effective antibacterial performance against MDR-infected wounds. Additionally, a recent study demonstrated that AgNPs-immobilized Schiff-base macrocycles as POD-mimicking nanozymes could be prepared via an in situ reduced approach. The Ag/Schiff-base nanozymes exhibited strong catalytic antibacterial activity with good biocompatibility and were further applied in antibacterial films [31]. Over all, metal nanozymes show high catalytic activity, tunable enzyme-like functions, and strong stability, enabling effective antimicrobial applications. However, issues such as cytotoxicity, metal ion release, and long-term biosafety remain concerns for clinical translation.

#### 2.1.2. Metal Oxide Nanozymes

Strong enzyme-mimetic properties, particularly CAT- and POD-like activity, are displayed by metal oxide nanoparticles including Fe_3_O_4_ [32], CeO_2_ [33], Mn_3_O_4_ [34], and CuO [35]. Their catalytic efficiency is impacted by surface imperfections, oxygen vacancies, and shifting oxidation states. For instance, CeO_2_ nanozymes alternate between Ce^3+^/Ce^+^ redox states, exhibiting both pro-oxidative and antioxidative behavior depending on the microenvironment [36]. Additionally, Fe_3_O_4_ nanozymes participate in Fenton or Fenton-like reactions, generating hydroxyl radicals (•OH) with potent biomedical properties [37]. These features make metal oxide nanozymes particularly useful in diseased wound situations with elevated H_2_O_2_ levels. Metal oxide nanozymes exhibit enzyme-like catalytic activity, low-cost fabrication, high stability, and multifunctional biomedical applications. However, challenges such as cytotoxicity, uncontrolled ROS generation, limited substrate selectivity, and metal ion release remain barriers to safe clinical translation.

#### 2.1.3. Carbon-Based Nanozymes

Carbon-based nanozymes such as graphene oxide (GO) [15], carbon quantum dots (CQDs) [38], and graphene-supported metals/metal oxides [39] offer improved biocompatibility, tunable surface chemistry, and intrinsic catalytic activity. Their different functional groups promote strong interactions with bacterial membranes and biofilms and also improve dispersibility. When paired with metal nanoparticles, carbon materials can significantly boost catalytic activities and enhance electron-transfer efficiency. Because of their versatility and minimal cytotoxicity, carbon-based nanozymes are widely employed in hydrogels, dressings, and polymeric scaffolds for wound-repair applications [40]. Single-walled carbon nanotubes (CNTs) were shown to exhibit POD-like activity [41], and they are now widely explored in antibacterial applications. Bamboo-like nitrogen-doped CNTs encapsulating cobalt NPs were developed by He et al. [42] as OXD-mimicking nanozymes. These nanozymes generated ROS under acidic conditions, enabling effective antibacterial activity against *S. aureus* and *E. coli* and promoting infected wound healing. Guo et al. [43] developed a GO-supported bimetallic copper-iron sulfide (CuFeSx) nanozyme that exhibits acid-responsive POD-like activity and photothermal enhancement, enabling localized ROS release in an infected microenvironment. Their study also proposed that synergistic catalytic and near-infrared (NIR) light-triggered ROS generation effectively eradicated methicillin-resistant *Staphylococcus aureus* (MRSA) and biofilms while accelerating infected wound healing. Carbon-based nanozymes exhibit potential enzyme-like activity, high biocompatibility, good chemical stability, and cost-effective synthesis. However, limitations such as restricted substrate selectivity, inconsistent catalytic efficiency, and a limited understanding of structure–activity relationships remain.

#### 2.1.4. Metal-Organic-Framework-Based Nanozymes

Metal ions combined with organic ligands generate the porous crystalline solids known as metal-organic frameworks (MOFs) [44]. Their large surface area, tunable pore structure, and excellent metal exposure make them attractive platforms for the development of multifunctional nanozymes. MOF-based nanozymes can offers synergistic antibacterial and ROS-regulating characteristics by combining catalytic metal centers such as Fe, Cu, or Mn with bioactive ligands [45]. MOFs are ideal for combined therapies in complex wound healing systems because they also allow for controlled therapy drug loading and release. Recently, Yang et al. [46] reported a highly conjugated two-dimensional copper, with hexahydroxy triphenylene (HHTP)–MOF nanozymes, featuring non-coordination saturated Cu sites that enable cascaded enhancement of POD-like activity. Their synergistic effects of strong H_2_O_2_ adsorption and photo-assisted electron transfer promoted efficient •OH generation, resulting in potent antibacterial activity. MOF-based nanozymes offer enzyme-mimetic activity, high porosity, and tunable active sites for multifunctional applications. However, instability in physiological environments, metal ion release, biocompatibility concerns, and scalability challenges limit clinical translation.

#### 2.1.5. Two-Dimensional Nanozymes

Two-dimensional (2D) nanozymes such as ultrathin nanosheets and layered nanostructures have emerged as highly effective platforms for antibacterial and wound-healing applications owing to their exceptionally large specific surface area, abundant exposed active sites, and accelerated interfacial electron transfer [47]. These features facilitate close contact with bacterial membranes and biofilm matrices, thereby enhancing catalytic-like activities and promoting efficient ROS generation. In particular, the planar geometry and atomic-scale thickness of 2D nanozymes enable enhanced catalytic accessibility and mass transport compared with their bulk counterparts, resulting in improved antibacterial efficacy at lower dosages [48].

Recent studies have demonstrated that 2D nanozymes can effectively disrupt bacterial membranes through combined physical contact and catalytic ROS-mediated oxidative stress, leading to rapid bacterial inactivation. Moreover, several 2D nanozyme systems exhibit photothermal or photo-enhanced catalytic behavior, allowing synergistic antibacterial action under NIR irradiation [49], which is particularly advantageous for infected wound environments. Moreover, 2D nanozymes have also been shown to regulate inflammatory responses and promote angiogenesis, thereby supporting tissue regeneration and accelerated wound closure [48].

#### 2.1.6. Single-Atom Nanozymes

SAzymes represent an advanced class of enzyme-mimetic catalysts in which isolated metal atoms are atomically dispersed on suitable supports, such as carbon matrices, metal oxides, or two-dimensional substrates [50]. By maximizing the atomic utilization efficiency and providing uniform, well-defined coordination environments, SAzyme exhibit superior catalytic activity, selectivity, and stability compared with nanoparticle-based systems, while simultaneously minimizing metal loading and potential toxicity [51].

In antibacterial applications, SAzyme demonstrate highly efficient peroxidase- and oxidase-like activities, enabling sustained ROS production even under the low H_2_O_2_ concentrations typically present in infected wound microenvironments. This high catalytic efficiency allows SAzyme to achieve potent antibacterial effects at ultra-low dosages, which is particularly beneficial for chronic and diabetic wounds where biosafety is a critical concern [52]. Furthermore, the tunable coordination chemistry of single-atom sites enables rational regulation of redox activity, inflammatory modulation, and macrophage polarization, contributing to improved wound healing outcomes [53].

### 2.2. Enzyme-Mimetic Activities

The capacity of nanozymes to mimic the catalytic activity of natural enzymes while providing improved stability, durability, and tunability is the main factor driving their medicinal applications. Nanozymes exhibit strong activity in a variety of physiological conditions, in contrast to biological enzymes, which are frequently sensitive to pH, temperature, and denaturing environments [12]. Their enzyme-like functions are determined by a combination of structural characteristics, metal valence states, surface electron-transfer kinetics, and lattice defects. These processes are essential for antibacterial activity, biofilm disruption, oxidative stress management, and microenvironment modulation in the wound healing.

POD-like catalysis enables the nanozyme-mediated conversion of H_2_O_2_ into highly reactive •OH radicals and is among the most extensively studied catalytic mechanisms [54]. By causing damage to bacterial membranes, proteins, and DNA, these radicals have potent bactericidal effects. Strong POD-like activity is demonstrated by metal and metal oxide nanozymes, especially those based on Fe, Cu, Mn, and noble metals, such as Pt and Au [55]. These nanozymes are particularly useful in infected wounds with elevated endogenous H_2_O_2_ activity, which allows them to catalyze the reduction of oxygen to produce ROS, even in the absence of H_2_O_2_. Particularly powerful OXD mimics are noble metals like Au, Ag, and Pt, which continuously produce ROS for excellent biofilm breakdown and broad-spectrum antibacterial action [56].

CAT-like activity enables nanozymes to decompose H_2_O_2_ into O_2_ and H_2_O, representing another crucial catalytic function [57]. CAT-like activity both reduces excessive oxidative stress and increases oxygen availability in hypoxic wound tissues, whereas POD-like behavior raises ROS levels to kill pathogens. This double action is advantageous for lowering inflammation, protecting nearby healthy cells, and encouraging angiogenesis. Similarly, by changing superoxide anions into oxygen and H_2_O_2_, superoxide dismutase (SOD)-like activity helps regulate oxidative equilibrium. Strong SOD-like effects that shield fibroblasts, keratinocytes, and other wound-healing cells are made possible by the reversible Ce^3+^/Ce^4+^ redox cycling of materials like cerium oxide (CeO_2_) [58].

Certain nanozymes, especially those made of Au, Pt, or MOF hybrids, also display glucose oxidase (GOx)-like activity [59]. These substances have the ability to oxidize glucose to develop H_2_O_2_, which then influences antimicrobial processes akin to those of POD [60]. For a diabetic wound setting with high glucose concentrations, this is particularly beneficial. Moreover, some nanozymes exhibit catalytic properties similar to those of DNase, allowing them to cleave extracellular DNA [61,62]. These nanozymes impair microbial defenses, improve antibiotic penetration, and lessen chronic inflammation linked to persistent infections by upsetting the structural integrity of the biofilm matrix.

## 3. Metal Functionalization Strategies

The nanoscale engineering of nanozymes, especially metal functionalization has a significant impact on their therapeutic efficacy. Metal-functionalization techniques are intended to boost stability, increase catalytic activity, improve antibacterial efficacy, and facilitate wound-microenvironment-responsive behavior [63]. Surface engineering, metal doping or decorating, biopolymer composting, and integration into hybrid platforms like hydrogels and nanofibers are some ways nanozymes are used in wound-healing applications by precisely controlling redox activity, ROS formation, ion release, and interactions with bacterial cells or biofilms [64].

### 3.1. Size and Morphology Regulation

Size control remains a fundamental regulatory strategy for tuning nanozyme catalytic activity, as the particle size directly governs the surface-to-volume ratio, active site exposure, and catalytic kinetics. Reducing nanozyme dimensions generally increases the number of accessible catalytic sites and accelerates electron transfer, resulting in enhanced enzyme-like activities [11,65]. However, excessively small nanozymes may suffer from aggregation or rapid clearance, underscoring the importance of balanced size optimization. Morphology regulation, including the design of nanosheets, nanodots, or porous nanostructures, further improves substrate accessibility and bacterial membrane contact, thereby strengthening antibacterial efficacy under physiological wound conditions [66,67].

### 3.2. Surface Ligand and Interface Engineering

Surface engineering is a popular method for enhancing the bioactivity and biocompatibility of nanozymes [68]. In biological settings, adding polymers like polyethylene glycol (PEG) [69], polyvinylpyrrolidone (PVP) [70], and chitosan (CS) [71] to the nanozyme surface can increase the circulation time, decrease aggregation, and improve colloidal stability. The selectivity and potency of the nanozyme catalytic effects can be increased through surface functionalization with peptides, antimicrobial compounds, or thiol-containing ligands, which can also enable targeted attachment to bacterial membranes or biofilm matrices [72]. An extra degree of control over the nanozyme interactions with the wound microenvironment is made possible by these surface changes.

### 3.3. Metal Doping and Decoration

By adding more metal components to the nanostructures, metal doping and decorating techniques increase the catalytic potential of nanozymes. [73] Through synergistic redox interactions, adding metals like Ag, Cu, Au, Pt, Zn, or Mn can greatly increase POD-, OXD-, or CAT-like activities. Ag–Cu [74], Au–Pt [75], and Zn–Fe [76] combinations are examples of bimetallic systems that can outperform single-metal nanozymes in terms of catalytic efficiency because of better electron transport, a higher surface defect density, and adjustable oxidation states. These combined effects boost the nanozymes’ capacity to breakdown biofilms and control oxidative stress in wound tissues, in addition to increasing antibacterial efficacy. The decoration of nanozymes with noble metals like Au or Pt introduces photothermal functionality, which allows synergistic photothermal and catalytic antibacterial effects [51].

### 3.4. Polymeric Composites and Hybrid Functionalization

The additional degree of adaptability is introduced by the incorporation of metal-functionalized nanozymes into polymeric carriers. Nanozymes can be included into hydrogels, nanofibers, films, or injectable matrices to assist the control of their release, enhance the local retention, and create a scaffold that supports tissue regeneration [77]. Wound dressings that can distribute metal nanozymes in a regulated and sustained manner are frequently made using hydrogels based on CS, gelatin, alginate, or polyvinyl alcohol (PVA) [78]. These composite systems offer oxygen permeability, moisture retention, and exudate absorption-qualities necessary for the best possible wound healing. With their high surface area, adjustable porosity, and effective interaction with wound surfaces, electrospun nanofibers combined with metal nanozymes improve the antibacterial efficacy while lowering the risk of infection [79].

### 3.5. MOF-Based and Stimuli-Responsive Metal Functionalization

Using MOFs as platforms for metal-functionalization is another new approach. Because of their extremely porous structure, MOFs enable the integration of medicinal compounds, catalytic ligands, and metal ions into a single framework [80]. This is because their modular design, catalytic activity, release kinetics, and structural stability may all be precisely adjusted. Multi-enzyme biomimetic activity and synergistic antibacterial effects can be demonstrated using MOF-based nanozymes, which are frequently loaded with Fe, Co, or Mn. MOF-nanozymes are great options for intelligent wound dressings because they provide advanced functions like pH sensitivity, glucose responsiveness, and stimuli-triggered activation when paired with polymers or biomolecules [64,81].

Another effective strategy to increase the versatility of nanozymes is stimuli-responsive metal functionalization [64]. Researchers can develop nanozymes that selectively activate in infected or chronic wound conditions by creating metal-functionalized systems that react to acidic pH, elevated H_2_O_2_, high glucose levels, or external stimuli like NIR light [82]. This tailored activation maximizes tissue-healing results and antibacterial activity while reducing possible cytotoxicity.

## 4. Antibacterial Mechanisms of Metal-Functionalized Nanozymes

**Figure 2:**
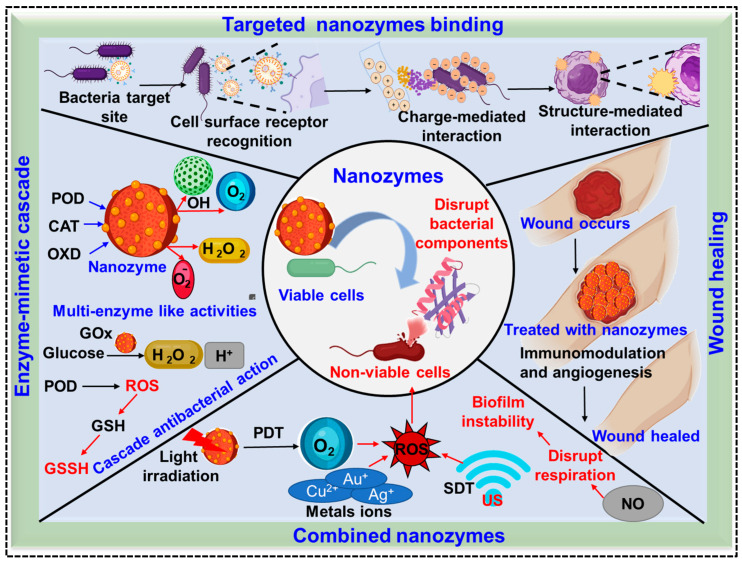
Schematic representation of targeted and combined metal-functionalized nanozymes for antibacterial and wound healing. Nanozymes bind bacteria through receptor-, charge-, and structure-mediated interactions and exhibit multi-enzyme-like activities (POD, CAT, OXD, and GOx) to produce ROS, disrupt redox homeostasis, and induce bacterial death. Synergistic PDT, SDT, and metal ion release and NO further destabilize biofilms, while immunomodulation and angiogenesis promote wound healing.

Metal-functionalized nanozymes eradicate pathogenic bacteria and promote wound repair through a coordinated combination of targeted bacterial interactions, enzyme-mimetic catalysis, and multimodal synergistic therapies. These antibacterial effects operate through a dual-mode mechanism, consisting of (i) redox enzyme-mimetic catalysis-driven ROS generation and (ii) metal/metal ion-mediated antibacterial actions, which act synergistically to disrupt bacterial membranes, biofilms, and intracellular metabolic processes [16]. Figure 2 schematically illustrates the antibacterial and wound-healing mechanisms of targeted and combined metal-functionalized nanozymes. Initially nanozymes selectively bind to bacterial target sites through cell surface receptor recognition, charge-mediated electrostatic attraction, and structure-mediated physical interactions, enabling effective localization at bacterial membranes and biofilm matrices [83]. Following targeted localization, nanozymes exert multi-enzyme-like activities including all oxidase functions, which catalyze the conversion of endogenous H_2_O_2_ into highly reactive •OH leading to damage to the bacterial cell membrane, proteins, and nucleic acids. In addition, GOx-like activity generates H_2_O_2_ and protons, developing a locally acidic microenvironment that further enhances POD-mediated ROS production [84,85].

The enzyme-mimetic cascade also induces glutathione (GSH) depletion and conversion to GSSG, disrupting bacterial redox homeostasis and amplifying oxidative stress, thereby converting viable bacterial cells into non-viable cells [86,87]. In the combined nanozyme strategy, catalytic antibacterial effects are synergistically enhanced by photodynamic therapy (PDT), sonodynamic therapy (SDT) triggered by ultrasound (US), and controlled metal ion releases (Ag^+^, Cu^+^, or Au^+^), which collectively promote ROS generation, biofilm inhibition, membrane disruption, and the inhibition of bacterial respiration. The additional release of nitric oxide (NO) further interferes with bacterial metabolic process and biofilm integrity [88]. For example, Feng et al. [89] demonstrated that a copper nitrite reductase-mimicking nanozyme is capable of dynamically tuning NO production in response to the infection microenvironment, thereby enabling both effective MDR bacterial elimination and subsequent tissue regeneration.

Beyond antimicrobial action, nanozymes actively regulate the wound microenvironment by scavenging excess ROS, suppressing prolonged inflammation, promoting macrophage polarization toward a pro-healing phenotype, enhancing angiogenesis, and stimulating ECM remodeling [85,90]. Although metal-based nanozymes primarily kill bacteria via ROS generation, their selectivity arises from microenvironment-responsive activation in infected wounds, which are characterized by an acidic pH and elevated H_2_O_2_ levels. Under normal physiological conditions, nanozyme activity is markedly reduced, minimizing damage to healthy tissues. Moreover, many nanozymes exhibit CAT- and superoxide dismutase-like activities during later healing stages, enabling excess ROS scavenging, inflammation suppression, and the promotion of tissue regeneration and angiogenesis. Through this integration of infection control, immunomodulation, and tissue regeneration, metal -functionalized nanozymes provide a comprehensive and effective strategy for accelerating wound healing in infected and chronic wound settings. The following subsections provide detailed bacterial inhibition mechanism of metal-functionalized nanozymes.

### 4.1. Direct Bactericidal Mechanisms

Metal-functionalized nanozymes display significant direct bactericidal activity through a combination of catalytic and physicochemical interactions that effect bacterial survival. One of the main mechanisms of action is the catalytic production of ROS such as H_2_O_2_, singlet oxygen, and superoxide anions, which cause oxidative damage to proteins, nucleic acids, and bacterial membranes [49,91,92]. These ROS species impair the function of metabolic enzymes, cause lipid peroxidation, damage membrane integrity, and ultimately cause cellular collapse. The release of antimicrobial metal ions interfaces with vital biochemical pathways, binds to thiol groups in enzymes, and hinders electron transport processes necessary for energy production, all of which contribute to bacterial death in addition to ROS-driven effects [93]. The strong interactions with the bacterial cell wall are also made possible by the nanoscale size and large surface area of metal nanozymes, which cause membrane instability and physical deformation. When combined, these mechanisms, metal ion toxicity, membrane rupture, and catalytic ROS production, allow for rapid and effective bactericidal activity, especially against antibiotic-resistant bacteria, which are frequently seen in chronic wound dressings. For example, Xiao et al. [94] demonstrated that the developed Ag/MoS_2_ nanozyme hydrogel showed remarkable antibacterial activity against *E. coli* and *S. aureus* by generating ROS to damage bacteria cells, while its porous structure effectively trapped and confined the bacteria.

### 4.2. Antibiofilm Mechanisms

In addition to the elimination planktonic bacteria, metal-functionalized nanozymes have strong antibiofilm properties that tackle one of the main issues with persistent wound infections. The EPS, composed of mainly proteins, polysaccharides, and extracellular DNA (eDNA), forms the structural matrix of biofilms, providing bacteria with structural defense and increasing their resistance to drugs [95]. Because of the small size and functionalized surfaces, nanozymes can efficiently penetrate biofilm matrices and facilitate catalytic activity right inside the biofilm microenvironment. ROS produced by nanozymes degrade cell–cell adhesion and damage EPS components and the structural integrity of biofilm networks [96]. Furthermore, eDNA, a crucial scaffold material for biofilm stabilization, is catalytically cleaved by nanozymes with DNase-like activity. A multinuclear Ce(IV)-based nanozyme effectively inhibited biofilm formation, dispersed mature biofilms, and enhanced antibiotic efficacy due to its high stability, deep biofilm penetration, and magnetic recyclability, outperforming natural DNase in antibiofilm applications [62]. By disturbing quorum sensing pathways, metal ions generated by nanozymes further impede bacterial communication and stop biofilm development and recycle processes. Metal-functionalized nanozymes are highly effective in dismantling established biofilms and stopping their reconstruction on wound surfaces through the combined actions of ROS-mediated EPS degradation, DNase-like cleavage, biofilm penetration, and quorum sensing preparation.

### 4.3. Immunomodulatory Effects

Metal- functionalized nanozymes have direct antibacterial and anti-biofilm properties, but they also significantly influence the host immune response, which is crucial for re-establishing regular wound-healing processes. Nanozymes assist in maintaining oxidative equilibrium in wound tissues by controlling ROS levels through enzyme-like activities [16,83]. This reduces excessive inflammation, which frequently impedes the healing of chronic wounds. In order to facilitate tissue regeneration, collagen deposition, and angiogenesis, some metal nanozymes such as those based on Cu, Ce, or Au encourage macrophage polarization from the pro-inflammatory M1 phenotype to the pro-healing M2 healing. For example, an injectable oxidized hyaluronic acid and carboxymethyl chitosan (OHA/CMCS) hydrogel loaded with copper-tetra(4-carboxyphenyl) porphyrin-manganese (Cu-TCPP-Mn) nanozymes effectively modulated the inflammatory microenvironment of infected wounds. The nanozyme system regulated excessive ROS levels through enzyme-like antioxidant activity, thereby alleviating prolonged inflammation. Importantly, it promoted macrophage polarization from the pro-inflammatory M1 phenotype toward the pro-healing M2 phenotype, accompanied by enhanced angiogenesis, collagen deposition, and the sustained release of growth factors from PRP [97]. A comparative overview of reported antibacterial nanozymes including their catalytic activities and mechanisms of action are provided in Table 1.

Overall, the table reveals that the antibacterial performance of metal-functionalized nanozymes is closely linked to their redox catalytic activity, composition, and structural design. Systems combining ROS-mediated catalysis with metal-ion-assisted antibacterial effects consistently demonstrate enhanced biofilm inhibition and therapeutic potential.

## 5. Metal-Specific Nanozyme Platforms for Wound Healing

**Figure 3:**
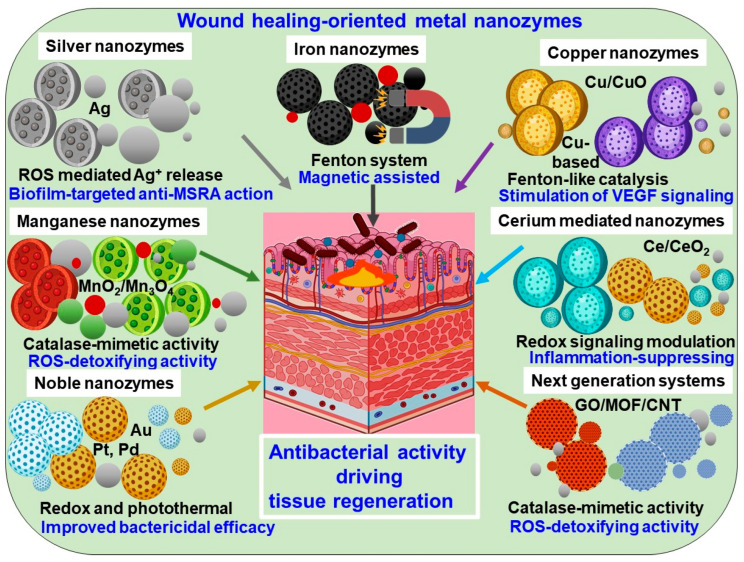
Metal-specific nanozyme platform for wound healing. The schematic summarizes major wound-healing metal nanozymes. These nanozymes exhibit metal-dependent enzyme-mimetic activities such as Fenton/Fenton-like catalysis, ROS generation or detoxification, redox modulation, and photothermal enhancement. Collectively, they eradicate pathogenic bacteria, regulate inflammation, promote angiogenesis, and accelerate tissue regeneration.

Metal-specific nanozyme platforms are a rapidly developing class of multifunctional materials that combine tissue-regenerative properties with catalytic antibacterial activity [13]. The catalytic behavior, biological interactions, and therapeutic results of nanozyme-based wound therapies are significantly influenced by the choice of metal type. Different redox characteristics, enzyme-mimetic activities, and biological effects are reported for each metal, allowing for customized methods of treating diabetic, chronic, and infected wounds. According to recent studies, metal-based nanozymes are positioned as next-generation wound-healing treatments since they not only kill harmful bacteria but also control inflammation, improve angiogenesis, and speed up tissue remodeling [116]. Figure 3 provides an overview of wound-healing-oriented metal nanozymes, illustrating their metal-specific enzyme-mimetic activities, antibacterial mechanisms, and roles in tissue regeneration. The following subsections discuss each metal-based nanozyme platform in detail, highlighting their distinct catalytic behaviors, biological functions, and therapeutic relevance in wound healing.

### 5.1. Silver-Based Nanozymes

**Figure 4:**
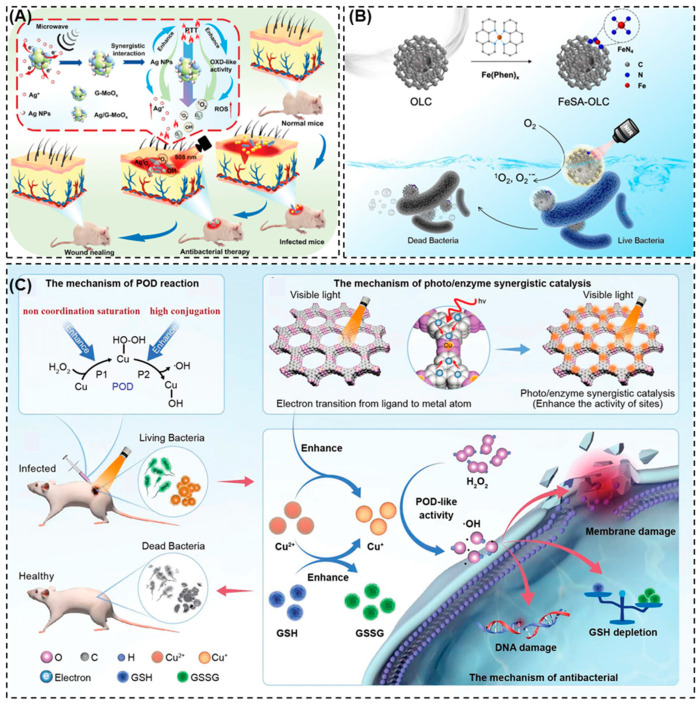
Mechanistic pathway of nanozyme-driven antibacterial activity and infected wound repair. (**A**) Schematic diagram showing the Ag/G-MoO_X_ synergistic antibacterial action against MDR bacteria. (**B**) Fe single-atom-decorated onion-like carbon nanozyme showing OXD-like activities under visible light, producing ROS that kill pathogens. (**C**) Schematic diagram of POD-reaction process and mechanism of synergistic catalysis and antibacterial action. Reproduced with permission from Zhang et al. [120] (Copyright, 2024, Wiley-VCH GmbH), Feng et al. [122] (Copyright, 2025, Elsevier) and Yang et al. [46] (Copyright, 2024, Wiley-VCH GmbH).

The most researched antibacterial platforms for wound healing are Ag-based nanozymes such as metallic Ag, silver oxide (Ag_2_O), and Ag-doped metal oxides [117]. These nanozymes rapidly and sustainably develop ROS at wound sites through potent POD- and OXD-like activities [118]. A synergistic mechanism involving both catalytic ROS generation and regulated Ag ion release gives Ag-nanozymes their antibacterial activity [119]. Ag ions further disrupt metabolic enzymes, interfere with DNA replication, and weaken membrane integrity, while ROS induce oxidative damage to bacterial membranes and intercellular components. When it comes to multidrug-resistant organisms like *Pseudomonas aeruginosa*, *Staphylococcus aureus*, and methicillin-resistant *Staphylococcus aureus* (MRSA), which often form persistent biofilms in chronic wounds, this dual-mode action is especially effective. Furthermore, Ag-based nanozymes show outstanding compatibility with hydrogel and dressing matrices, allowing for regulated ion release to minimize cytotoxicity and maintain the antibacterial activity [16]. Recently, Zhang et al. [120] developed an Ag-decorated glucose-modified molybdenum oxide (MoOx) as a reductive support for in situ AgNPs deposition (Figure 4A). The introduction of Ag potentially improved NIR photothermal conversion and OXD-like catalytic activity and enabled NIR-accelerated Ag^+^ release. This synergistic combination of hyperthermia, ROS generation, and controlled Ag^+^ delivery resulted in the effective eradication of MRSA and promoted the accelerated healing of infected wounds [121].

### 5.2. Iron-Based Nanozymes

Iron-based nanozymes, such as Fe_3_O_4_ nanoparticles and iron-containing MOFs are well known for their capacity to catalyze Fenton reactions that produce extremely reactive •OH [123]. These radicals cause DNA damage, protein oxidation, and lipid peroxidation, resulting in potent bactericidal activity. Iron-based nanozymes are especially suitable for wound healing due to their sensitivity to the high H_2_O_2_ levels found in pathological tissues [83]. For instance, an onion-like carbon-supported single-atom iron nanozyme (FeSA-OLC) was developed by Feng et al. [122], which combines OXD-like catalysis with photothermal activity for antibacterial applications (Figure 4B). The atomically dispersed Fe-N_4_ sites efficiently activate molecular oxygen to generate ROS without external H_2_O_2_, while NIR further enhances catalytic efficiency. This synergistic photothermal catalytic effect results in improved antibacterial performance. Fe_3_O_4_ nanozymes offer antibacterial effects and magnetic responsiveness for enhanced localization at wound sites. Additionally, by catalytically breaking down H_2_O_2_ into oxygen, iron-based nanozymes can aid in wound oxygenation by reducing hypoxia and promoting angiogenesis. Iron nanozymes are exceptionally effective at treating infected and chronic wounds because of their combination of antibacterial action, targeted delivery, and microenvironment manipulation [124]. Nie at al. [125] reported a polymerization-induced self-assembly (PISA)-derived ultrasmall Fe-based nanozyme for the non-antibiotic treatment of infected wounds. The nanozyme exhibits POD-, CAT-, and GPx-like activities, along with NIR photothermal properties, enabling efficient ROS generation and GSH depletion in acidic infection microenvironments. Combined with 808 nm irradiation, this synergistic catalytic–photothermal therapy eradicated 98% of MRSA in vivo and markedly accelerated wound healing, promoting re-epithelialization and collagen regeneration.

### 5.3. Copper-Based Nanozymes

Based on their strong redox cycling and Fenton-like catalytic activity, Cu-based nanozymes, such as Cu, Cu_2_O, CuO, and copper-based MOFs, have become extremely effective catalytic systems [84]. These nanozymes provide broad-spectrum antibacterial activity against both Gram-positive and Gram-negative bacteria by efficiently producing ROS under physiological circumstances. A study by Yang et al. [46] developed a highly conjugated 2D Cu-HHTP MOF nanozyme with non-coordination saturated Cu sites to derive a cascaded, enhanced POD-like reaction for antibacterial applications (Figure 4C). The unsaturated Cu centers improve H_2_O_2_ adsorption, while the conjugated framework promotes visible-light-assisted electron transfer jointly boosting •OH generation. As a result, the nanozyme achieved 99.99% bacterial elimination at a low dose, exhibited potential biofilm disruption, and accelerated infected wound healing with reduced inflammatory cytokines and good biocompatibility. Copper nanozymes are essential for wound healing via biological signaling pathways, in addition to their antibacterial qualities. It has been demonstrated that the release of Cu^2+^ ions increases vascular endothelial growth factor (VEGF), which promotes angiogenesis and enhances the development of blood vessels in injured tissues [18,126]. In ischemic and diabetic wounds where vascularization is compromised, this pro-angiogenic impact is especially advantageous. Additionally, copper-based nanozymes have outstanding chemical and thermal durability, which allows for sustained therapeutic action and catalytic activity under a variety of wound-healing conditions. Tian et al. [127] developed a copper–gallic acid (Cu-GA) coordination polymer nanozyme with triple enzyme-like activities for treating bacteria-infected wounds. The Cu-GA nanozyme exhibits POD- and GSH-Px-like activities under acidic conditions, which enable efficient ROS generation and glutathione depletion to kill *E. coli* and MRSA, while showing SOD-like activity under neutral conditions to scavenge excess ROS and reduced inflammation. In vivo studies demonstrated accelerated wound closure, a reduced bacterial load, suppressed inflammatory cytokines, and enhanced angiogenesis without noticeable toxicity.

### 5.4. Manganese-Based Nanozymes

Manganese-based nanozymes, such as MnO_2_ and Mn_3_O_4_, are characterized by their potent capacity to control oxidative stress and their pH-responsive CAT-like activity [128]. Mn-based nanozymes reduce oxidative damage and alleviate hypoxia in acidic and inflammatory wound settings by breaking down hydrogen peroxide into oxygen and water [85]. A manganese–gallic acid (MnGA) nanozyme was prepared, with dual SOD- and CAT-like activities, by Guo et al. [129] for acute wound healing by alleviating oxidative stress and inflammation. MnGA efficiently scavenges ROS and reactive nitrogen species (RNS) through a cascade antioxidant mechanism, suppresses pro inflammatory cytokines, and accelerates wound closure in vivo. A transcriptomic analysis revealed the modulation of nuclear factor (NF)-κB, toll-like receptor (TLR), and NOD like receptor (NLR) signaling pathways.

For diabetic wounds, where high ROS buildup and a poor oxygen supply impede healing, this dual capability is especially beneficial. Manganese nanozymes shield surrounding tissues and encourage cell migration, proliferation, and ECM deposition by scavenging dangerous ROS and re-establishing the redox balance [130]. Depending on the wound environment, their adaptable catalytic behavior allows them to act as both tissue-protective and antibacterial agents. For instance, Li et al. [90] reported an ultra-small Mn_3_O_4_ nanozyme with dual SOD- and CAT-like activities for diabetic wound healing. The prepared nanozyme efficiently scavenges excess ROS to suppress chronic inflammation, enabling macrophage polarization from the pro-inflammatory M1 state to the pro-healing M2 phenotype. This immune regulation enhances angiogenesis, collagen deposition, and re-epithelization, leading to significantly accelerated wound closure in diabetic mice without observable toxicity.

### 5.5. Cerium-Based Nanozymes

Based on the reversible Ce^3+^/Ce^2+^ redox switching, which allows for the dynamic modulation of oxidative stress during various stages of wound healing, cerium (Ce) nanozymes are active because of their redox flexibility, (Ce) nanozymes can scavenge excess ROS and act as antioxidants in inflammatory tissues [131]. When needed, they can also have pro-oxidant antibacterial activity. Recently, Filippova et al. [132] systematically investigated the POD-like activity of CeO_2_ nanozymes and demonstrated that both the particle size and chemical environment critically govern their catalytic performance. Using chemiluminescent assays, the authors showed that smaller CeO_2_ nanoparticles exhibit distinct POD-like behavior depending on the buffer composition, with phosphate ions significantly modulating surface reactivity and H_2_O_2_-decomposition kinetics. Their findings highlight that CeO_2_ nanozyme activity is not solely size-dependent but strongly influenced by surface hydroxylation, Ce^3+^/Ce^4+^ redox states, and surrounding chemical environments, underscoring the importance of reaction conditions when designing Ce-based nanozymes for biomedical applications such as antibacterial therapy and wound healing. Another study by Yuan et al. [133] developed an ultrasmall Ce-MOF nanozyme with strong hydrolase-like activity for antibiofilm therapy. The key mechanism arises from the synergistic Ce (IV)/Ce (III) redox couple and abundant surface Ce-OH Lewis acid sites, which cooperatively activate water and phosphate/glycosidic bonds. These disrupt EPS, which leads to biofilm destabilization. CeO_2_ nanozymes exhibit potent actions similar to those of superoxide dismutase and CAT, shielding fibroblasts and keratinocytes from oxidative damage and encouraging tissue repair. CeO_2_ nanozymes minimize collateral tissue damage while promoting angiogenesis, improving collagen deposition, and regulating inflammation to aid in coordinated wound repair [134]. They are especially useful for chronic and non-healing wounds because of their capacity to regulate oxidative and antioxidative activities.

### 5.6. Noble Metal Nanozymes

Platinum, gold, and palladium are examples of noble metal nanozymes that have remarkable catalytic efficiency because of their robust electron-transfer capacity and high surface activity [135]. Even at low doses, ROS-mediated antibacterial activity is made possible by these nanozymes’ strong OXD- and POD-like capabilities. While Pt nanozymes provide improved endurance under challenging circumstances, Pt-based nanozymes are especially valued for their stability and wide-spectrum catalytic efficacy [136]. Apart from their antibacterial qualities, Au-based nanozymes have demonstrated exceptional promise in stimulating angiogenesis and tissue regeneration [137]. A bimetallic nanocluster enzyme (Au/Pt NCs) designed with high POD-like catalytic activity chemically coupled with GOx showed potent antibacterial activity against *F. nucleatum* and their biofilms [100]. Noble metal nanozymes can also be used in conjunction with photo thermal treatment, which uses the synergistic thermal and oxidative effects of near-infrared radiation to increase catalytic activity [13]. This photo thermal–catalytic combination provides a potent method for getting rid of resistant germs and speeding up wound healing.

### 5.7. Emerging Metal Systems

The new nanozyme platforms that go beyond traditional metals are attracting interest for use in wound healing. Because zinc-based nanozymes have good biocompatibility and inherent antibacterial activity, they can be used as topical wound dressings [138]. When utilized as substrates for metal nanozymes, two-dimensional materials like MoS_2_ boost surface contacts and electron transport, increasing catalytic efficiency [139]. In order to maximize catalytic performance while reducing metal usage and toxicity, isolated metal atoms are anchored onto support materials in SAzymes or a bi- and tri-metallic complex [140]. These technologies provide excellent enzyme-mimetic performance and precise control over active sites, creating new opportunities for the development of safe and highly effective nanozyme-based wound treatments.

## 6. Metal–Polymer Composite Nanozyme Dressings

Incorporating metal-functionalized nanozymes into polymeric wound dressings has proven to be a highly effective method of combining biological and structural support for tissue regeneration with catalytic antibacterial activity [141]. In addition to serving as carriers that stabilize and regulate the release of nanozymes, polymer matrices also perform vital wound-healing tasks such as mechanical protection, moisture retention, and exudate absorption. Composite dressings that include nanozymes into biocompatible polymers can provide long-lasting antibacterial action while also supporting angiogenesis, cell proliferation, and ECM modeling [142]. The following subsections provide a detailed discussion on important polymer-functionalized metal nanozymes for wound-healing applications.

### 6.1. Chitosan-Based Nanozyme Dressings

CS-inherent antibacterial qualities, biocompatibility, biodegradability, and superior wound adherence make CS-based nanozymes dressings one of the most researched composite systems. Because of the polycationic nature of CS and the catalytic activity of the embedded nanozymes, these composites show synergistic antibacterial effects when mixed with metal nanozymes [143]. Metal nanozymes produce ROS and release the metal ions, increasing their antibacterial activity, while CS breaks down bacterial membranes through electrostatic interactions [119,144]. A surface-engineered CS-coated iron-based MOF (MIL)-53 (Fe) nanozyme exhibits dual POD- and OXD-like activities through biomimetic catalytic mechanism. Mechanistically, the Fe^3+/^Fe^2+^ redox centers in MIL-53 (Fe) serve as the important catalytic sites, while CS introduces abundant -NH2 and -OH groups that form hydrogen bonds with H_2_O_2_, facilitating O-O bond cleavage and •OH radical generation, analogous to histidine-assisted catalysis in horseradish POD [145].

**Figure 5:**
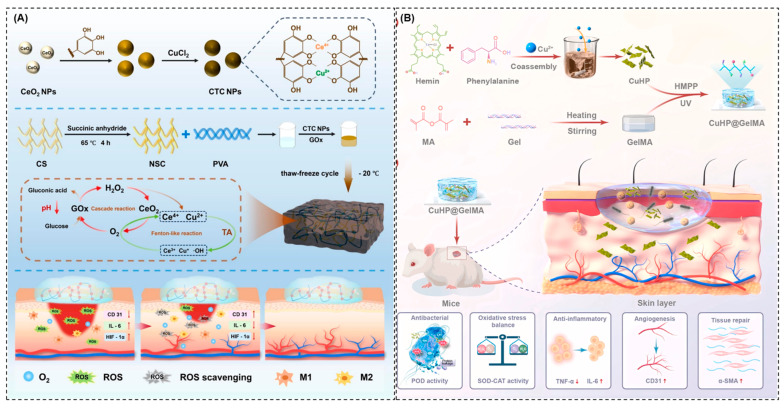
(**A**) Schematic diagram of NP hydrogel preparation and mechanism of CeO_2_ nanozyme synergistic GOx dual pathway in the treatment of diabetic wounds. (**B**) Schematic illustration of the anti-inflammatory and antibacterial effects mediated by CuHP@GelMA for enhanced wound healing. Reproduced with permission from Ge et al. [147] (Copyright, 2026, Elsevier), and Wang et al. [148] (Open access, Copyright, 2026, Elsevier).

Additionally, CS offers a flexible framework for creating hydrogels that are injectable, sticky, and self-healing. These hydrogels can retain close contacts with the wound bed and conform to uneven wound geometrics [146]. These characteristics reduce systemic exposure, allow for the localized and prolonged administration of nanozymes, and produce a moist, protective environment that speeds up tissue regeneration and wound healing. For example, Ge et al. [147] reported a nanozyme-doped CS hydrogel for diabetic wound healing, where CS serves as a biocompatible scaffold that stabilizes and retains nanozymes at the wound site (Figure 5A). The authors designed a multifunctional system in which CeO_2_-tannic acid–Cu (CTC) nanozymes are embedded within a succinylated CS/PVAA hydrogel. The coordinated incorporation of Ce and Cu ions within the nanozyme framework provides multi-enzyme mimetic activity including CAT-, SOD-, and Fenton-like functions. Embedding these nanozyme into a CS-based hydrogel ensures mechanical stability, wound adherences, and sustained retention, while allowing efficient diffusion of glucose and reactive species. The designed nanozyme system responds dynamically to diabetic wound cues. Excess glucose is enzymatically converted into H_2_O_2_, which is subsequently regulated by CeO_2_ redox cycling to generate oxygen and scavenge excess ROS, alleviating hypoxia and oxidative stress. Under acidic, infected conditions, Cu-mediated Fenton-like reactions selectively produce bactericidal radicals, enabling effective infection control without prolonged oxidative damage. Further in vivo diabetic wound models demonstrated markedly improved wound-closure rates, reduced inflammatory markers, and enhanced vascularization with no evident systemic toxicity. CS-based nanozyme dressings provide strong antibacterial synergy, good biocompatibility, and sustained localized delivery for wound healing. However, limitations such as mechanical instability, degradation-related variability, and potential metal-ion-associated cytotoxicity remain concerns.

### 6.2. Gelatin- and Collagen-Based Dressings

Gelatin- and collagen-based nanozymes are particularly attractive for wound healings due to their ECM-mimetic nature [149]. Collagen and its derivative gelatin provide structural prompts that facilitate cell adhesion, migration, and proliferation, which are critical for effective wound repair [150]. The incorporation of metal nanozymes into these protein-based scaffolds enhances their antibacterial function without compromising their bioactivity. The catalytic activity of nanozymes within collagen or gelatin matrices enables localized ROS generation to eliminate bacteria, while the ECM-like framework supports fibroblast infiltration, angiogenesis, and tissue remodeling. A gelatin methacryloyl (GelMA)-based CuHP nanozyme hydrogel (CuHP@GelMA) was developed by Wang et al. [148] for accelerated wound-healing applications (Figure 5B). The developed GelMA network localizes and stabilizes the CuHP nanozymes, ensuring uniform distribution and sustained activity at the wound site by controlling substrate diffusion and ROS exposure, and gelatin enables efficient early-stage POD-like antibacterial catalysis followed by a transition to ROS-scavenging activity as healing progresses. These dressings are especially beneficial for chronic wounds where impaired cell migration and delayed matrix formation hinder healing. Additionally, collagen-based composites can be cross-linked to improve mechanical stability and degradation control, ensuring prolonged therapeutic efficacy [151,152]. Gelatin- and collagen-based nanozyme dressings provide ECM-mimetic support, promote cell adhesion and tissue regeneration, and enable localized antibacterial activity. However, challenges such as rapid degradation, limited mechanical strength, and potential instability of embedded nanozymes may affect long-term clinical performance.

### 6.3. Alginate and PVA-Based Dressings

Alginate and PVA are two polymers that are frequently used in wound care because of their superior ability to absorb exudate and their mechanical durability [153,154]. When alginate-based nanozyme dressings come into touch with wound exudate, they quickly produce hydrogels that kill pathogens and excess fluids while promoting healing. Long-term antibacterial activity at the wound site is ensured by the controlled and sustained release of catalytic agents, made possible by the incorporation of metal nanozymes into alginate matrices. A metal-based alginate nanozyme system was developed by Yang et al. [155]. The designed nanozyme showed the distinct catalytic roles of Au and Fe_3_O_4_. Au nanoparticles act as GOx-like nanozymes under alkaline conditions, generating H_2_O_2_ from glucose, while Fe_3_O_4_ exhibits POD-like activities under acidic conditions, converting H_2_O_2_ to reactive oxygen species via Fe^2+^/Fe^3+^ cycling. The alginate hydrogels ionically crosslinked with Ca^2+^ spatially separate and immobilize the metal nanozymes, prevent catalytic interfaces, and regulate mass transport and diffusion. Similar to this, PVA-based dressings offer strong flexible scaffolds that can withstand repeated mechanical stress without compromising the activity of nanozymes [156]. Advanced dressings that balance fluid control, antibacterial action, and tissue protection can be developed by combining alginate or PVA with metal nanozymes. These dressings are especially useful for severally oozing or infected wounds. Alginate and PVA-based nanozyme dressings offer effective exudate management, good mechanical strength, and sustained antibacterial activity. However, challenges such as excessive swelling, diffusion-related variability, and potential instability of catalytic performance may limit long-term therapeutic effectiveness.

### 6.4. Electrospun Nanofibrous Mats

Metal nanozyme-loaded electrospun nanofibrous mats are a well-developed class of wound dressings that closely resemble the fibrous structure of the ECM of native skin [157]. Electrospun fibers’ high surface-area-to-volume ratio enables effective nanozyme loading and close contact with wound surfaces, improving antibacterial activity [158,159]. Broad-spectrum antibacterial effects and synergistic catalytic activity are made possible by the introduction of multi-metal nanozymes into nanofibers [160]. Additionally, controlled oxygen diffusion and moisture regulation are made possible by the porous structure of electron-spun mats, both of which are critical for the possible wound healing. These mats can be designed to facilitate cell infiltration and tissue regeneration while offering long-lasting antibacterial activity by adjusting the fiber diameter, porosity, and nanozyme composition [161]. Electrospun nanozyme-loaded nanofibrous mats closely mimic the native ECM, provide a high surface area, and support sustained antibacterial and regenerative functions. However, limitations such as complex fabrication, mechanical fragility, and uneven nanozyme distribution may restrict their long-term clinical use.

## 7. Performance in Wound-Healing Models

The standardized in vitro tests and physiologically appropriate in vivo wound models are necessary for assessments of metal-functionalized nanozymes for wound healing. The efficacy of nanozymes must be evaluated in infected and inflammatory wound microenvironments since their catalytic mechanisms rely on variables like the pH, hydrogen peroxide concentration, oxygen availability, and local biomolecular interactions. Preclinical testing often aims to measure catalytic ROS-related behavior, validate biofilm-eradication efficiency, confirm broad-spectrum antibacterial activity, and guarantee cytocompatibility with important wound-healing in different cell types. Together, these investigations determine whether nanozyme-based treatments may promote tissue regeneration and eradicate infection at the same time without causing intolerable toxicity.

### 7.1. In Vitro Tests

The minimum inhibitory concentration (MIC) and minimum bactericidal concentration (MBC) against pertinent wound pathogens, such as *S. aureus*, MRSA, *P. aeruginosa*, and other opportunistic bacteria are usually the first step in in vitro antibacterial testing [162,163,164]. These tests offer quantifiable standards for assessing the efficacy of nanozymes in different formulations and for fine-tuning elements including the dose, surface functionalization, and metal composition. An antibiofilm assessment is equally important since wound infections are often linked to biofilms [165]. In contrast to eradication experiments, which often include biomass staining, viable cell counting, and microscopy-based visualization, biofilm-inhibition assays evaluate the efficacy of nanozymes to prevent biofilm development. ROS detection tests are frequently used to verify and measure the creation of reactive species since catalytic ROS generation is a key antibacterial mechanism for metal nanozymes [166,167]. Depending on the nanozyme system and stimulus circumstances, 1, 3-Diphenylisobenzofuran (DPBF) is often employed to assess singlet oxygen-related activity [168], while probes like 2, 7-Dichlorodihydofluorescein (DCFH-DA) are commonly utilized to monitor intracellular or bulk ROS production [169]. Fibroblasts and keratinocytes, which are essential for wound closure and re-epithelialization are frequently used in cytocompatibility testing since antibacterial efficacy must be balanced with safety. Assays for cell viability, proliferation, migration, and membrane integrity are utilized to ensure that nanozymes and their polymer matrix do not impede tissue-repair mechanisms or cause excessive oxidative damage.

### 7.2. In Vivo Wound Models

The most significant evaluation of metal-functionalized nanozyme results in therapeutic effects under intricate physiological settings comes from in vivo research. Diabetic wounds represent a clinically significant condition characterized by reduced angiogenesis, chronic inflammation, elevated oxidative stress, and delayed tissue remodeling. Diabetic mouse wound models are commonly employed to evaluate therapeutic efficacy [85]. Nanozyme-based dressings have demonstrated effectiveness in accelerating wound closure, reducing microbial burden, and restoring the normal healing process in these models.. The practical potential of nanozyme systems to manage highly persistent wound infections and biofilms can be further tested using infection models with MRSA or *P. aeruginosa* [16,121]. Bacterial colony counting, histopathology, inflammatory cytokine analysis, and wound-closure kinetics are used in these models to evaluate the data. Angiogenesis and tissue remodeling indicators are used to assess regeneration outcomes beyond infection control. To measure neovascularization and determine if the dressing enhances the vascular density and perfusion at the wound site, CD31 immunostaining is frequently utilized. Mason’s trichrome staining, which offers information on ECM remodeling and scar quality, is commonly used to assess collagen and structural restoration. Monitoring markers like CD86 for pro-inflammatory M1 macrophages and CD206 for pro-healing M2 macrophages is often used to study macrophage polarization because immunological dysregulation is a significant cause of chronic wound persistence [170,171]. Reduced inflammation, increased granulation tissue development, and better wound healing are typically linked to nanozyme systems that encourage a shift towards M2-dominant responses [172]. Overall, these in vivo outcomes validate both the antibacterial efficacy and the ability of metal-functionalized nanozymes to establish a balanced environment that facilitates effective and superior tissue regeneration. A comparative summary of commonly used in vitro antibacterial assays and in vivo wound-healing models for evaluating metal-functionalized nanozymes is presented in Table 2.

## 8. Characterization Techniques for Metal-Based Nanozymes

Physicochemical characterization is fundamental for elucidating the enzyme-mimetic behavior of metal-based nanozymes and correlating their structural features with antibacterial performance. Fourier-transform infrared spectroscopy (FTIR) is commonly used to confirm surface functional groups and metal–ligand coordination, which influence substrate adsorption and catalytic efficiency in redox-enzyme-like reactions [173].

Nanozyme catalytic activity is strongly dependent on the particle size, morphology, and defect density, which are routinely analyzed using scanning electron microscopy (SEM) and transmission electron microscopy (TEM) [174]. High-resolution TEM further provides insights into lattice fringes, crystal defects, and surface vacancies that serve as active sites for ROS generation and redox cycling [24]. Atomic force microscopy (AFM) is employed to evaluate surface roughness and topography, particularly for nanozyme-coated films and hydrogels used in wound healing [175].

X-ray diffraction (XRD) is widely applied to identify crystalline phases associated with enzyme-like activity [176], while X-ray photoelectron spectroscopy (XPS) reveals metal oxidation states and surface chemistry that govern redox-driven catalysis [177,178]. UV–visible spectroscopy is frequently used to monitor nanozyme–substrate interactions and reaction kinetics [23], whereas electron paramagnetic resonance (EPR) spectroscopy enables the direct detection of enzymes such as POD and OXD, providing mechanistic evidence for ROS-mediated antibacterial activity [179].

In addition, dynamic light scattering (DLS) and zeta potential analysis are routinely used to assess the hydrodynamic size, colloidal stability, and surface charge, which are critical for bacterial targeting, biofilm interactions, and selective antibacterial action [22]. Together, these complementary techniques provide a comprehensive understanding of structure–activity relationships and support the rational design and optimization of nanozyme-based antibacterial and wound-healing systems.

## 9. Safety, Biocompatibility, and Toxicity Considerations

Metal-functionalized nanozymes have intriguing antibacterial and regenerative properties, but their practical translation necessitates the need for assessments of long-term toxicity, safety, and biocompatibility. The unregulated release of metal ions such as Ag^+^, Cu^2+^, or Zn^2+^, which can have deleterious effects on nearby healthy tissues when present at high concentrations, is one of the main difficulties with metal-based nanozymes [115]. Although metal ion release plays a major role in the effectiveness of antibacterial agents, excessive exposure can interfere with normal cellular processes, reduce the survival of fibroblasts and keratinocytes, and delay the healing the wounds [180]. Thus, striking a compromise between cytocompatibility and antibacterial potency continues to be a crucial design problem.

The excessive production of ROS is another significant safety hazard. ROS are necessary for killing bacteria and breaking up biofilms, but excessive or prolonged ROS generation can harm host tissues by causing DNA damage, protein oxidation, and lipid peroxidation [181,182]. In a sensitive wound environment like diabetic or ischemic tissues, chronic oxidative stress may worsen inflammation, hinder angiogenesis, and impede tissue regeneration [183,184]. In order to maintain ROS levels within a therapeutic level that supports antibacterial action without generating collateral tissue damage, nanozyme systems must be properly designed.

Potential risks include the long-term accumulation and persistence of metal-based nanozymes in tissues, particularly those composed of non-biodegradable materials. Metals that have accumulated may cause systemic distribution outside of the wound site, disrupt regular cellular metabolism, or cause chronic inflammatory reactions [185,186]. This highlights the importance of understanding nanozyme-degradation pathways, clearance processes, and bio-distribution profiles, especially for recurrent or long-term wound treatments. Therefore, thorough in vivo toxicity studies are necessary to assess both systemic and local effects over long time periods.

Numerous engineering techniques have been developed to increase the biocompatibility of metal-functionalized nanozymes in order to address these safety concerns. CS, PEG, gelatin, and alginate are examples of polymer coatings that are frequently used to control metal ion release, lessen direct metal–cell interaction, and enhance overall cytocompatibility. Another crucial strategy is surface charge regulation, as a positive charge might boost antibacterial activity but also increase cytotoxicity. By adjusting the surface charge, nanozymes can interact with bacterial membranes efficiently while causing the least amount of harm to mammalian cells.

Biodegradable matrices improve nanozyme safety by enabling post-therapy degradation and clearance [78,187]. Lastly, controlled ROS production and redox behavior appropriate to certain wound microenvironments are made by precisely adjusting enzyme-mimetic activity through surface engineering, metal doping, or ligand modification [188]. When combined, these approaches are essential for creating metal-functionalized nanozyme systems for wound healing applications that are safe, efficient, and therapeutically transferable.

## 10. Clinical Translation and Commercial Potential

The clinical translation of metal-functionalized nanozyme-based wound therapies is supported by the long-standing use of metallic materials in commercial wound care products, particularly Ag- and Cu-containing dressings that have received regulatory approval for antimicrobial applications. These products have demonstrated effective infection control in a wide range of clinical settings, providing a strong precedent for the incorporation of metal-based technologies into wound management. Building on this foundation, nanozyme platforms offer significant advantages over conventional metallic dressings by introducing catalytic, self-sustaining antibacterial activity, microenvironment-responsive behavior, and multi-functional therapeutic effects. Although most nanozyme systems remain at the preclinical stage, increasing numbers of studies have demonstrated their efficacy and safety in animal wound models, highlighting their potential for future clinical evaluation.

Despite its potential, a number of issues need to be resolved before nanozyme-based wound dressings are widely used in clinical settings. Since many nanozyme-synthesis techniques require complicated processes, stringent reaction conditions, or expensive materials that could impede large-scale production, scalability continues to be a major constraint. For commercialization and regulatory approval, uniform catalytic performance and batch-to-batch reproducibility are essential [189,190]. Translation is made more difficult by biosafety issues, such as metal ion release, long-term accumulation, and possible off-target effects, which call for thorough toxicological testing. Furthermore, the classification of nanozymes, whether as pharmaceuticals, medical devices, or combination products can have a substantial impact on approval processes and development schedules, and regulatory frameworks for nanozymes based treatments are currently developing.

In the future, a viable avenue for commercialization leads to the incorporation of nanozymes into wearable therapeutic devices and smart wound patches. Intelligent dressings that react dynamically to changes in the wound microenvironment can be made by integrating metal-functionalized nanozymes with biosensors that can monitor the wound pH, temperature, glucose levels, or infection indicators [191,192]. These technologies could optimize the therapeutic dose, activate catalytic antibacterial properties on demand, and give physicians real-time feedback. These developments fit in nicely with the expanding trend of individualized and precise wound treatment.

The development of nanozymes and their clinical translation are anticipated to be accelerated by artificial intelligence (AI) and computational techniques [193]. To reduce the need for trail-and-error testing, the ideal metal composition, surface architectures, and catalytic sites can be found using density functional theory (DFT) simulations, machine learning techniques, and predictive catalysis models [194]. AI-driven methods have the potential to increase productivity, reduce development costs, and improve the safety and effectiveness of nanozyme-based wound treatments by facilitating rational design and performance prediction. All of these development points to metal-functionalized nanozymes being in a good position to move from experimental platforms to commercially feasible and clinically significant wound-healing therapies in the near future.

## 11. Limitations

Despite the rapid progress in metal-functionalized nanozymes for antibacterial wound healing, several limitations remain. Most current studies are largely restricted to in vitro experiments and small-animal models, and their long-term therapeutic efficacy and safety in clinically relevant wound settings are not yet fully established. In particular, potential concerns related to metal ion release, prolonged ROS exposure, immunogenicity, and tissue accumulation require systematic long-term evaluations.

Another limitation lies in the lack of standardized synthesis protocols and evaluation criteria, which complicates direct comparisons of catalytic activity, antibacterial efficacy, and biocompatibility across different nanozyme platforms. Variations in wound models, bacterial strains, and activity assays further hinder reproducibility and translational consistency. Additionally, challenges associated with large-scale production, batch-to-batch uniformity, and regulatory classifications remain significant barriers to clinical adoption. Addressing these limitations through standardized testing frameworks, long-term in vivo studies, and scalable manufacturing strategies will be critical for advancing nanozyme-based wound therapies toward practical clinical applications.

## 12. Future Directions

The fast development of metal-functionalized nanozymes has created new possibilities for the development of more accurate, flexible, and biologically integrated next-generation antibacterial wound-healing treatments. SAzymes offer maximized atom efficiency and potent enzyme-mimetic catalysis while reducing the overall metal dosage [195]. Importantly, in wound-healing applications their primary value lies in minimizing long-term metal ion leaching and associated toxicity while maintaining effective antibacterial performance. Atomic-level control further provides unprecedented precision in modulating redox activity and antibacterial efficacy to suit distinct wound conditions [196].

Designing biofilm-specific nanozymes that target the structural and metabolic components of microbial biofilms is a critical research direction [197]. This requires moving beyond inherent catalytic activity toward conjugating nanozymes with targeting ligands or engineering microenvironment-responsive catalysts. Such systems can be tailored to degrade the EPS, interfere with quorum sensing pathways, or catalytically cleave eDNA rather than relying solely on nonspecific ROS generation. These strategies may help to overcome persistent biofilm-associated infections in chronic wounds and improve therapeutic outcomes through biofilm-targeted mechanisms. The additional step toward simulating intricate biological processes is the incorporation of multi-enzymatic cascade nanozymes [198,199]. The production and consumption of ROS in the wound microenvironment can be dynamically regulated by cascade systems that integrate the actions of GOx, POD, and CAT. Because high glucose levels can be used to initiate localized antibacterial activity and enzyme-like capabilities avoid excessive oxidative damage, such systems are especially appealing for diabetic wounds [200,201].

Injectable and stimuli-responsive hybrid hydrogels containing metal nanozymes are expected to play a central role in future wound therapies [82]. These materials react to external triggers like light or magnetic fields, as well as wound-specific stimuli, like the pH, H_2_O_2_ concentration, and glucose levels. Stimuli-responsive hydrogels improve patient comfort and treatment compliance while providing spatial and temporal precision through the on-demand activation and controlled release of nanozymes [202]. Additionally, their injectable nature enables intimate contact with uneven or deep wound sites and minimally invasive applications. The new area has been driven by developments in digital health and bio sensing technologies that are personalized for wound therapy. Real-time therapeutic activity modifications may be made possible by combining nanozymes with wearable biosensors that can track wound indicators like the pH, oxygen, glucose, and inflammatory mediators [203]. By enabling treatments to be customized to specific wound situations, disease states, and healing trajectories, these intelligent systems would ultimately increase the efficacy and minimize needle exposure to antimicrobial drugs.

Green and sustainable synthesis methods are expected to become increasingly significant in the advancement of nanozyme platforms [204]. Environmentally acceptable methods for producing metal nanozymes with improved biocompatibility and less ecological effect are provided by biomolecules sourced from marine and plant sources. By reducing the use of hazardous reagents and harsh processing conditions, these green synthesis techniques may aid in regulatory approval and are consistent with the principles of the circular bio economy [205]. Future nanozyme-based wound therapeutics have the potential to revolutionize advanced wound care by combining precise catalysis, smart responsiveness, personalization, and sustainability.

## 13. Conclusions

Metal-functionalized nanozymes represent a versatile class of catalytic materials that integrate enzyme-mimetic antibacterial activity, biofilm disruption, and wound microenvironment modulation within a single platform. Recently reviewed studies showed that these nanozymes consistently demonstrate the ability to catalytically generate ROS, release antibacterial metal ions, and interact directly with bacterial membranes and biofilm matrices, thereby addressing infection control as a primary barrier to wound healing. Furthermore, their catalytic stability and tunable redox behavior distinguish them from natural enzymes and conventional antimicrobial agents. From the pathophysiological perspective, chronic and non-healing wounds are characterized by persistent infection, dysregulated oxidative stress, prolonged inflammation, and impaired angiogenesis. The reviewed evidence indicates that metal-functionalized nanozymes can simultaneously target these interconnected challenges by achieving a rapid bacterial burden reduction, regulating ROS levels to avoid excessive oxidative damage, and activating pro-regenerative signaling pathways that support neovascularization, collagen deposition, and tissue remodeling. These multifunctional effects have been reported across diverse wound models, including diabetic and MDR infection settings, resulting in accelerated wound closure and improved tissue organization compared with non-catalytic controls.

Despite these advantages, several critical complications remain, including precise control over the catalytic intensity, long-term biosafety, metal ion accumulation, and batch-to-batch reproducibility. Recent advances reviewed herein such as SAzyme, defect and size engineering, stimuli-responsive activation, and integration into polymeric hydrogels and nanofibrous dressings represent promising strategies to overcome these limitations by enabling controlled, localized, and wound-adaptive catalytic activity. Overall, this review provides a structured and mechanism-driven framework linking nanozyme design principles to biological function and therapeutic outcomes. By critically evaluating current progress alongside existing challenges, it offers practical guidance for the rational development of safer, more effective, and application-specific nanozyme-based platforms for advanced wound care.

## Figures and Tables

**Table 1:** Summary of representative nanozyme-based antibacterial nanoplatforms highlighting their enzyme-mimetic activities, targeted pathogens, and primary antibacterial mechanisms.

Nanoplatforms	Nanozyme Activity	Pathogens	Mechanism of Action	Reference
Pt-MoS_2_	POD, OXD	*E. coli*, *S. aureus*	Pt-MoS_2_ showed enhanced POD- and OXD-like activities and GSH depletion under NIR irradiation, leading to efficient antibacterial activity.	[98]
PT-Ru NCs	POD	*E. coli*, *S. aureus*	Pt-Ru showed enhanced POD-like activity, promoting •OH generation from H_2_O_2_.	[99]
Au/Pt NCS@GOx	POD, Gox	*F. nucleatum*	Au/Pt NCS@GO_X_ achieved effective bacterial inhibition through glucose-triggered cascade catalytic •OH generation in situ.	[100]
GOx-CaPCuPt	POD, OXD, CAT	*E. coli*, *S. aureus*	GOx-CaPCuPt showed POD/CAT-like activity with synergistic CDT-PTT sterilization.	[101]
Mo/Fe_2_@CuO_2_	POD	*E. coli*, *S. aureus*	Mo/Fe_2_@CuO_2_ promoted GSH depletion and H_2_O_2_ self-supply, boosting NIR-driven antibacterial activity.	[102]
MoS_2/_CoS_2_	POD, OXD	*E. coli*, *S. aureus,* MRSA	MoS_2_/CoS_2_ enhanced POD/OXD-like activity via accelerated interfacial electron transfer.	[103]
MoS_2_@CP	POD	*E. coli*, MRSA	NIR irradiation enhanced the POD-like activity of MoS_2_@CP via acid-triggered H_2_O_2_ generation and Cu^2+^ release.	[104]
HM-CuO	POD	*E. coli*, *S. aureus*	HM-CuO nanozymes combined GSH depletion ability and PTT for enhanced antibacterial activity.	[105]
BiPt@HMVs	POD, OXD	MRSA	Ultrasound enhanced POD-like activity of BiPt@HMVs boosted antibacterial performance.	[106]
DA-PPI	POD	*E. coli*, *S. aureus*	DA-PPI nanozymes enhanced POD-like activity via integrating iridium (Ir)-modified Pd–Pt dendrimers.	[107]
CCS NPs	POD, OXD	*E. coli*, *S. aureus*	CCS NPs exhibited POD-like activity and GSH depletion of effective sterilization.	[108]
Ag@Pt nanozymes	POD	*E. coli*, *S. aureus*	Ag@Pt nanozymes showed enhanced POD-like activity via nucleic acid–metal ion coordination.	[109]
N-CNDs	OXD	*E. coli*, *S. aureus*	Light-activated OXD-like N-CNDs enabled synergistic antibacterial ability.	[110]
CNQDs	POD	*E. coli*, *S. aureus*, *B. subtilis*, *R. solani*	NV-rich CNQDS boosted POD-like activity through enhanced electron delocalization.	[111]
Cs@Fe/CDs	POD	*S. aureus*, *P. aeruginosa*	Cs@Fe/CDs enabled antibacterial effects through electrostatic binding and POD-like catalysis.	[112]
Co-Fe NDs	POD	MRSA	Size-downscaled Co-Fe NDs showed three-fold enhancement of POD-like activity	[113]
USAuNps/MOFs	POD	*E. coli*, *S. aureus*	US-AuNPs/MOF hybrid showed enhanced POD-like activity by integrating AuNPs and 2D MOFs.	[114]
Zn SACs@CuO_2_	POD	*E. coli*, *S. aureus*	Zn SACs@CuO_2_ combined high photothermal efficiency with Cu^2+^, PTT, and CDT for antibacterial action.	[115]

**Table 2:** Comparison of in vitro antibacterial assays and in vivo wound-healing models used to evaluate metal-functionalized nanozymes, highlighting key parameters and their translational relevance.

Evaluation Aspect	In Vitro Tests	In Vivo Wound Models
Primary objective	Assess antibacterial activity, ROS generation, and cytocompatibility.	Evaluate therapeutic efficacy and wound-healing performance under physiological conditions.
Common models	Bacterial cultures, biofilm assays, fibroblast/keratinocyte cell lines	Diabetic wound models, infected wound models (MRSA, *P. aeruginosa*).
Key parameters measured	MIC, MBC, biofilm inhibition, ROS production, cell viability, and migration.	Wound closure rate, bacterial burden, histopathology, angiogenesis, and collagen deposition.
Mechanistic insights	Redox catalytic activity, ROS-mediated killing, and cytotoxicity.	Tissue regeneration, immune modulation, angiogenesis, and ECM remodeling.
Techniques used	DPBF, DCFH-DA assays, microscopy, viability assays.	Histology, CD31 staining, cytokine analysis, and macrophage markers (CD86, CD206).
Translational relevance	Preliminary screening and optimization.	Preclinical validation of safety and therapeutic efficacy.

## Data Availability

No new data were created or analyzed in this study. Data sharing is not applicable to this article.
